# Research on Effects of Dust Removal Using Ultrasonic Vibrators

**DOI:** 10.3390/mi13122155

**Published:** 2022-12-06

**Authors:** Chong Li, Ruimin Chen, Da Gao

**Affiliations:** Research Center for Humanoid Sensing, Zhejiang Lab, Hangzhou 311100, China

**Keywords:** ultrasonic vibrators, dust removal, PZT plates, finite element

## Abstract

This work presents two types of ultrasonic vibrators in order to comparatively analyze their dust removal properties for microscopic particles. The vibrators were constructed by pasting four piezoceramic (PZT) plates onto the upper surface of a rectangular metal substrate. The longitudinal or bending mode is excited to form a standing wave in the vibrators. The superposition of the longitudinal and bending modes generates a traveling wave. Because the bending and longitudinal vibrations are two different modes, the process of tuning their resonant frequencies to be close is necessary for the traveling wave vibrator. The structural parameters of the vibrators were optimized by the finite element method. During experiments, the dust removal performances of these vibrators were evaluated by dumping cigarette ash or alumina powders randomly on the surface of the prototype vibrators. The measured experimental results indicate that the dust removal performance of the traveling wave oscillator is better than that of the standing wave oscillator. The two operating modes of the traveling wave vibrator produce orthogonal vibration displacements on the vibrator driving points. Vibration in one direction generates friction force, which drives adherent dust particles. Vibration in the other direction provides intermittent contact pressure between the vibrators and deposited dust particles. The synthesized elliptical motion of vibrator particles helps to improve the dust removal characteristics of the vibrators. The simple structure of the developed vibrators makes them the most promising candidates for dust removal from engines, camera lenses, car windows, and navigation systems.

## 1. Introduction

Oil contamination, the main cause of malfunctions in engine turbochargers, usually contains debris such as carbon build-up and swarf deposits. Although carbon and swarf deposits are small, when mixed with oil, they lead to wear and turbo failure. Besides this, fine dust particles, such as copper and aluminum powder, are produced during the welding process. If not treated in time, these deposited dust particles will affect the quality of the product, cause battery short circuiting, and pollute the workshop environment. Ultrasonic dust removal is one of today’s top cleaning methods with its high efficiency and eco-friendliness [1]. It is suitable for cleaning a variety of materials, including ceramics, metals, rubber, glass, and hard plastics. By using ultrasonic dust removal technology, tightly adhered microscopic particles ten to fifty microns in size can be removed from intricate items with blind holes, cracks, and recesses. Therefore, ultrasonic dust removal technology can be utilized to clean up fine dust particles from critical components in precise instruments, such as automobiles, engines, cameras, etc.

The basic principle of ultrasonic dust removal is that under the action of an ultrasonic wave, air produces fierce oscillation, and violent collision occurs between suspended dust particles, resulting in the condensation and settlement of dust particles [1]. Contaminants removed through ultrasonic cleaning include dust, grit, cigarette ash, swarf, silt, etc. It has been proven that ultrasonic waves can make tiny dust particles, which cannot be removed or are difficult to remove using water, settle down [1]. However, the frequency of the ultrasonic wave and dust concentration should be controlled well. In general, the appropriate frequency range of the ultrasonic wave is 25 kHz to 80 kHz. A higher frequency permits a higher level of intricate detail to be cleaned and results in gentler cleaning on parts.

An ultrasonic resonator consisting of exciting elements and a metal base is the key component in ultrasonic dust removal systems. Piezoelectric materials can convert electrical energy into mechanical energy through an inverse piezoelectric effect. Therefore, most ultrasonic cleaners adopt piezoelectric materials as the active element. Scholars have done much fruitful work in wet dust reduction technology [2]. Concretely, research on physical and chemical modification of water was carried out in order to improve wet dust reduction efficiency. However, there have been few studies on dry dust removal technology using ultrasonic resonators. Nowadays, although there are patents in the field of dry ultrasonic dust removal technology [3,4], its dust removal property and mechanism are still not quite clear. This paper studies the dry dust removal technology through utilizing specifically designed ultrasonic vibrators [5,6,7,8,9,10]. First, the structure and operating principle of the proposed ultrasonic vibrators are illustrated. Second, finite element simulation is carried out to acquire the dimensions of optimal ultrasonic vibrators. Finally, prototype vibrators were fabricated. Then, the impedance characteristics and dust removal performances were tested by means of experimental apparatus. The experimental results verified that the designed vibrators can clear away fine dust particles adhering to their surfaces. The presented ultrasound vibrators are simple in structure, easy to process, and low in cost. Moreover, these vibrators remove contaminant microparticles without additional cooling or heating processes. Therefore, they could well be applied in various fields of dust removal, especially in miniature high-end precision instruments.

## 2. Structural Design and Working Principle

**Figure 1:**
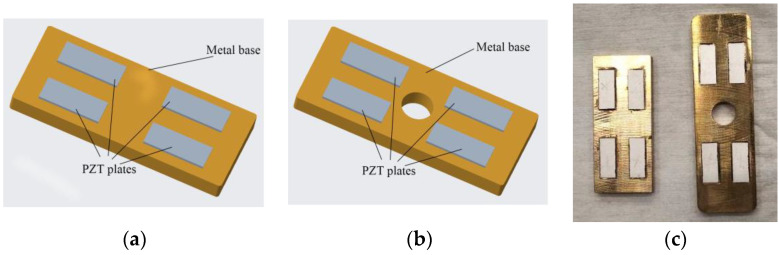
Three-dimensional structure of the vibrators: (**a**) The standing wave vibrator; (**b**) The traveling wave vibrator; (**c**) Prototype vibrators.

This paper exploits two kinds of vibrators, as shown in Figure 1, to comparatively analyze their dust removal performances. The three-dimensional structure of the standing wave vibrator is shown in Figure 1a. The traveling wave vibrator is indicated in Figure 1b. Figure 1c shows the prototype vibrators using the d_31_ effect of piezoelectric elements. These vibrators consist of a metal base and four PZT plates. The metallic component of the traveling wave vibrator is a single bronze plate with a center hole, whereas the metallic body of the standing wave vibrator is just a brass plate. PTZ plates were symmetrically bonded onto the top surface of a metal base using epoxy resin glue and polarized along the thickness direction. The positive pole surfaces of all the PZT plates are outward, and their negative pole surfaces are in contact with the metallic part that is connected to the ground. In order to accurately locate the paste positions of the PZT plates, thin grooves were cut in the area of the metal body where the PZT plates needed to be attached.

**Figure 2:**
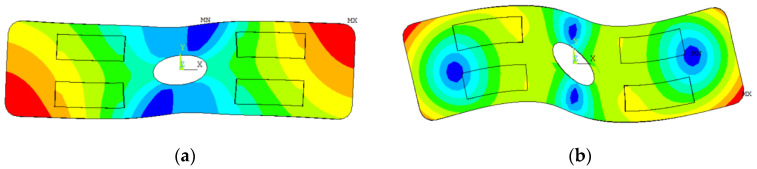
Operating modes of the traveling wave vibrator: (**a**) Longitudinal mode; (**b**) Bending mode.

The vibrator structure determines the existence of in-plane longitudinal and bending modes [10,11,12]. The first-order longitudinal mode and the second-order bending mode of the vibrators were selected as working modes. Specifically, a longitudinal or bending mode is excited by PZT plates, forming a standing wave in the vibrator. The traveling wave vibrator should combine two standing waves with a π/2 phase difference in both time and space. Therefore, in order to generate a traveling wave in the vibrator, the resonance frequencies of the longitudinal and bending modes need to be less than 200 Hz to achieve frequency consistency degeneracy. This means that the dimensions of the traveling wave vibrator should be carefully designed. However, frequency consistency degeneracy of the two vibration modes cannot be achieved by only adjusting the length, width, and thickness of the traveling wave vibrator. Accordingly, a hole was drilled in the metal base. In order to minimize the influence of the drilled hole on the vibration characteristics of the travelling wave vibrator, the hole should be located at the common vibration node of the two modes. Ultimately, the metallic plate of the traveling wave vibrator was perforated in the middle. The vibration modes of the traveling wave vibrator are displayed in Figure 2. The corresponding vibration modes of the standing wave vibrator are analogous.

**Figure 3:**
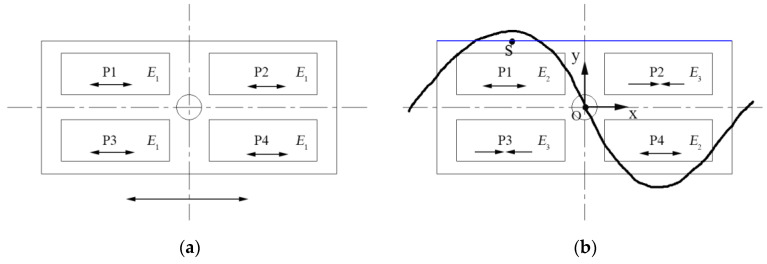
Vibration characteristics of the traveling wave vibrator under different vibration modes: (**a**) Longitudinal mode; (**b**) Bending mode.

The mode actuation mechanism of these ultrasonic resonators is similar. The traveling wave vibrator is taken as an example to analyze the excitation method of the longitudinal and bending modes. As shown in Figure 3, the four PZT plates are represented by P1, P2, P3, and P4. The metal base is grounded. When a sinusoidal AC voltage signal, *E*_1_ = *A*_L_ sin (2π*f*_L_*t*), is applied in the polarization direction of P1 to P4, the four PZT plates expand and contract in the horizontal direction simultaneously, as shown in Figure 3a. *A*_L_ and *f*_L_ are the amplitude and frequency, respectively, of the applied voltage. When the frequency of the excitation signal, *f*_L_, is equal to the resonance frequency of the longitudinal mode, the vibrator’s longitudinal resonance is stimulated. The four PZT plates repeat expansion and contraction, producing a standing wave pattern of the longitudinal mode inside the vibrator.

Figure 3b shows the second-order bending mode. To excite the bending mode, two AC voltages, *E*_2_ = *A*_B_ cos (2π*f*_B_*t*) and *E*_3_ = −*A*_B_ cos (2π*f*_B_*t*), should be applied to the four PZT plates according to Figure 3b. *A*_B_ and *f*_B_ are the amplitude and frequency, respectively, of the applied voltages. *f*_B_ is adjusted to the natural frequency of the bending mode. Under the bending mode, P1 and P4 elongate, whereas P2 and P3 shrink. The four PZT plates repeat elongation and shrinkage, producing a standing wave pattern of a bending wave inside the vibrator.

The combination of the two standing waves forms a traveling wave. In order to form a traveling wave in the vibrator, the same resonance frequency of the two working modes is needed. To excite two standing waves simultaneously in the vibrator, the voltages *E*_A_ = *A*_E_ sin(2π*f*_E_*t*) and *E*_B_ = *A*_E_ cos(2π*f*_E_*t*) are applied. Concretely, P1 and P4 are connected to excitation voltage *E*_A_. The other actuation voltage, *E*_B_, is applied to P2 and P3. *A*_B_ represents the amplitude of the applied voltages. *f*_E_ is the resonance frequency of the longitudinal and bending modes. The voltages have a temporal phase difference of π/2, while the two standing waves have a wavelength difference of π/2. Therefore, a traveling wave can be generated in the vibrator.

The longitudinal and bending modes provide horizontal and vertical deformations, respectively, of the vibrator particles. When dust grains are attached to the outer face of the vibrators, they definitely oscillate in response to the vibration of the vibrator particles. If the friction force *F* acting on a dust particle due to vibration activated by the PZT plates is larger than adhesion force *P*, relative sliding between the dust particle and vibrator occurs. Previous studies suggest that higher friction and lower adhesion forces are generated when the roughness of the contact surface between the vibrators and dust particles is higher. Then, dust is easier to remove from the vibrators. Therefore, the surface of the metal body without pasted PZT plates was roughened to improve dust removal performance.

**Figure 4:**
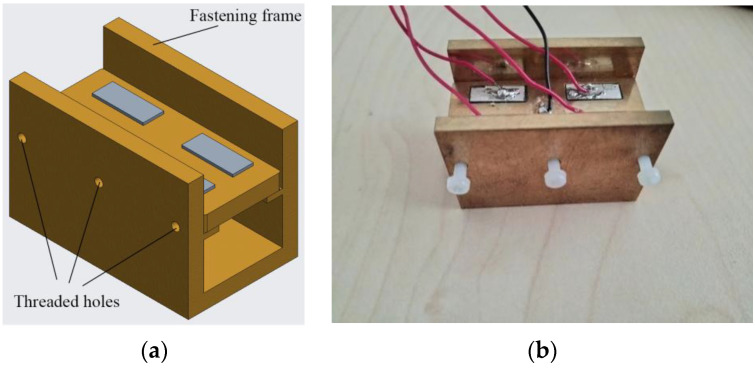
Structure of the clamp: (**a**) Three-dimensional model; (**b**) Actual machined support frame.

A fixture, as shown in Figure 4, was designed to adjust the position of the vibrators that can be illuminated by an electron microscope. Then, the observation region of the vibrators could also be regulated. The vibration behaviors of dusts along the vertical direction could be observed by changing the direction of the fastening structure’s placement reasonably [13]. Vibrators were placed on the supporting beam of the fastening structure. Flexible clamping is a critical factor in realizing an uninfluenced vibration mode and resonance frequency of the vibrators. Therefore, to keep the vibrators highly flexible, threaded shafts made of Teflon were used to apply pre-pressure on the vibrators. As can be seen from Figure 4, threaded holes were symmetrically arranged on both the left and right sides of the fastening architecture to freely select clamping positions. A horizontal preload was applied to the vibrator by the flexible thread shafts.

## 3. Simulations

The finite element method was used to visualize the vibration mode shape and estimate the natural frequency of the vibrators [14,15,16,17]. The vibration displacements of particles on the vibrator surface could also be calculated. The finite element model of the vibrators was established using ANSYS Parametric Design Language (APLD). The PZT plates used were the product PZT-47 of Shenzhen Sendils New Energy, Shenzhen, China. The dielectric, piezoelectric, and elastic matrices of the PZT plates are given in Table 1. The other material characteristics are those of hard piezoelectric material: density = 7.8 × 10^3^ kg/m^3^, Poisson’s ratio = 0.31, and mechanical quality factor = 1800. Brass was used as the metal parts for its higher hardness. The material characteristics are those of brass: Young’s modulus = 117 GPa, density = 8.9 × 10^3^ kg/m^3^, and Poisson’s ratio = 0.341.

Four PZT plates with a length of 10 mm, width of 4 mm, and thickness of 0.5 mm were bonded onto the top face of a metallic base to form the ultrasonic vibrators. The dimensions of the standing wave vibrator’s metal plate are listed in Table 2. L, W, and T respectively represent the length, width, and thickness. In order to tune the resonance frequencies of the longitudinal and bending modes of the traveling wave vibrator to be close, a metallic plate with a center hole was used. Further, by adjusting the structural parameters of the metal base, a certain traveling wave vibrator structure meeting the frequency degeneracy requirement was obtained. The optimal structural dimensions of the traveling wave vibrator’s metal plate are also listed in Table 2. D is the diameter of the center hole.

Through modal analysis, the unconstrained longitudinal and bending vibration modes of the proposed vibrators were obtained. The resonance frequencies of the longitudinal and bending vibration modes were also achieved and are listed in Table 3. The frequency difference between the two resonance modes of the traveling wave vibrator was only 80 Hz. From modal analysis, it was also concluded that there were no other interferential vibration modes near the longitudinal and bending modes.

Harmonic response analysis was performed to obtain the amplitude of the longitudinal and bending vibrations of particles on the vibrator surface at different frequencies. For the standing wave vibrator, a sinusoidal AC voltage with amplitude 100 V was applied to all PZT plates to excite the longitudinal mode, as illustrated in Figure 3a. When the resonance frequency was 51.15 kHz, the vibration displacement in the horizontal direction was the largest, at 18 µm. Similarly, when two sinusoidal waveform signals shifted by π were applied to the PZT plates, as shown in Figure 3b, the maximum vibration displacement of the vibrator surface particles in the vertical direction reached 14 µm.

**Figure 5:**
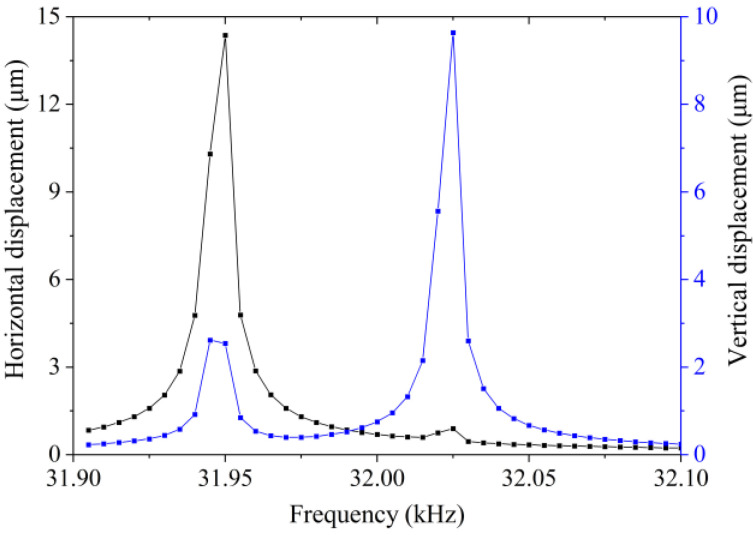
Vibration displacements of the observation spot under variant frequencies.

In order to simultaneously actuate the longitudinal and bending modes of the traveling wave vibrator, two sinusoidal AC voltages with a phase difference of π/2 and amplitude of 100 V were applied to the four PZT plates, as described in Section 2. We set the driving frequency range from 31.90 kHz to 32.10 kHz. The amplitude–frequency curve of a point on the vibrator surface in both the vertical and horizontal directions is given in Figure 5. The selected observation point is shown in Figure 3b, indicated by s. Its x, y, and z coordinates were −12 mm, 7.5 mm, and 0 mm, respectively. O, a point in the center of the hole, was considered as the origin of the coordinates.

**Figure 6:**
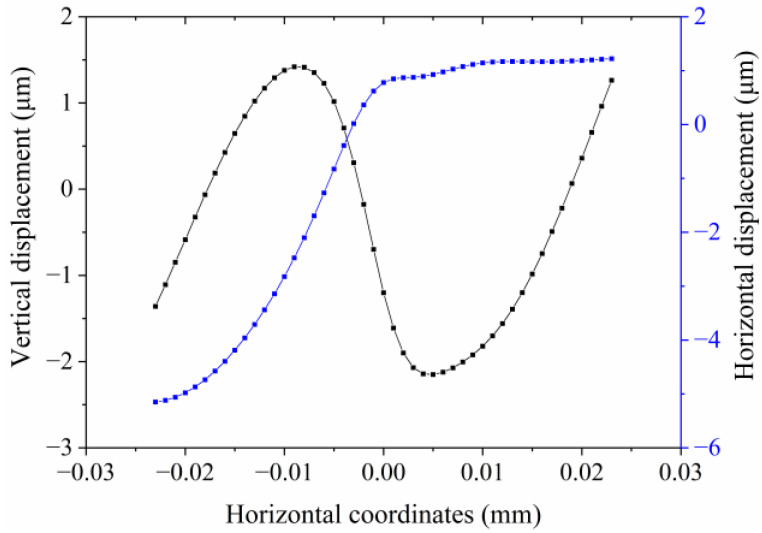
Amplitude characteristics of the chosen nodes.

Figure 6 exhibits the vibration displacements of the top surface nodes of the traveling wave vibrator under a 32 kHz excitation frequency. The chosen nodes were located at the blue line shown in Figure 3b. These nodes have the same y and z coordinates. The results are consistent with the shape curves of the exploited longitudinal and bending modes which can be deduced from Figure 2. The simulation results also show that the two operating modes can be simultaneously excited by using two driving voltages at a specific frequency of 32 kHz.

**Figure 7:**
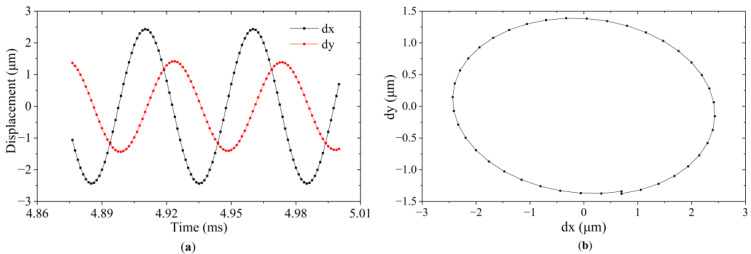
Vibration features of a vibrator driving point calculated by transient analysis at the frequency of 32 kHz: (**a**) Steady-state displacement; (**b**) Motion trajectory.

To investigate the motion trajectory of driving particles on the surface of the ultrasonic oscillators, the transient response was also analyzed. Based on the harmonic response analysis results, 32 kHz was selected as the driving frequency to simultaneously excite the two operating modes. The other parameters of the excitation voltages, such as amplitude and phase difference, were the same as those applied in the harmonic response analysis. The calculated displacement curves of the same observation point s under stable motion are shown in Figure 7a. dx and dy respectively represent the vibration displacement in the horizontal and vertical directions. The resultant vibration trajectory is shown in Figure 7b. As can be seen in Figure 7b, the vibration locus in the XY plane is elliptical in shape and can be controlled by adjusting the phase difference between the two actuation signals. Projection of the displacement trajectory to the X axis shows that it varies from −2.43 μm to 2.43 μm, while variation in the Y axis is from −1.38 μm to 1.38 μm. When the vibrator driving points and attached dust particles contact each other, friction force is generated and can be used as a driving force of the dust. Consequently, the vibration of the vibrator particles can drive the movement of dust particles attached to the vibrator surface.

## 4. Experiments

**Figure 8:**
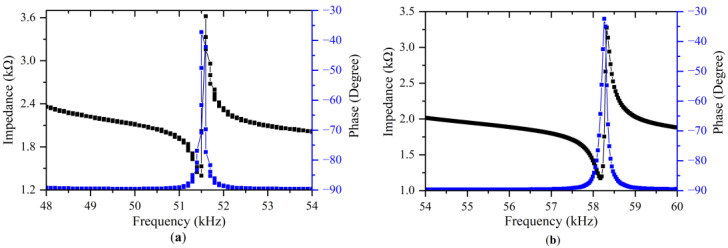
Admittance characteristics of the standing wave vibrator: (**a**) Longitudinal vibration; (**b**) Bending vibration.

The existence of resonant frequencies can be shown by measuring the change in impedance at various frequencies. When the vibrator resonates, its impedance reaches the minimum value. In order to measure the impedance characteristics of the vibrators, the signal and ground wires of an impedance analyzer (E4990A, Keysight Technologies Inc. CA, United States) were respectively connected to the top surfaces of the PZT plates and the metal base. The frequency response curves of the impedance and phase of the standing wave vibrator from 48 kHz to 54 kHz are shown in Figure 8a. A resonance was observed as the change in impedance at approximately 51.5 kHz. This change indicates that the resonance frequency of the longitudinal mode is 51.5 kHz for the standing wave vibrator. Figure 8b shows the admittance curve of the bending mode. As shown in Figure 8b, the resonance frequency of the bending mode is 58.2 kHz. The deviation percentages of the resonant frequencies obtained by simulation and experiment are displayed in Table 4.

**Figure 9:**
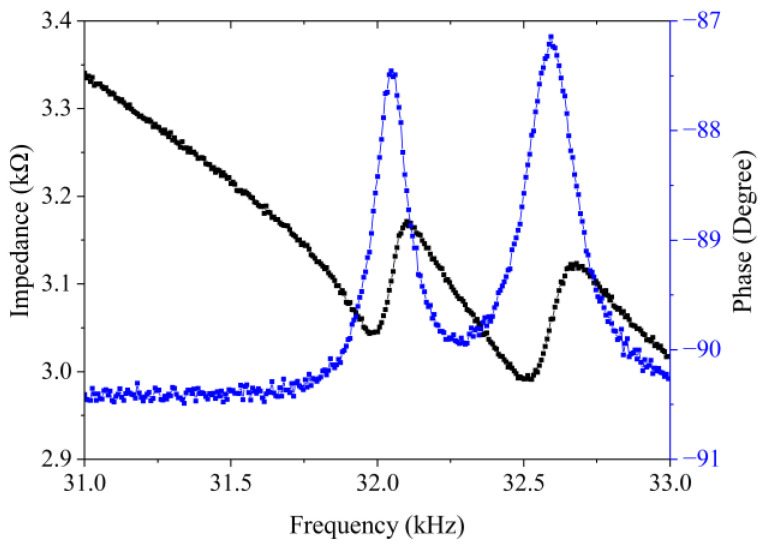
Frequency response characteristics of impedance and phase for the traveling wave vibrator measured using an impedance analyzer.

Figure 9 presents the admittance curve of the traveling wave vibrator. As shown in Figure 9, the resonance frequencies of the longitudinal and bending modes are, respectively, 32.07 kHz and 32.48 kHz. The frequency difference of these two modes is 0.41 kHz. There is a discrepancy between the experimental results measured by the impedance analyzer and the simulation results (see Table 4). This is caused by the epoxy resin glue used for pasting the PZT plates, which was omitted in the simulation analysis to simplify the model. Meanwhile, machining errors were inevitably produced during the actual manufacturing process of the vibrators, which also led to a larger frequency difference between the two vibration modes. Besides this, during experiments, the pre-pressure force exerted on the ultrasonic vibrators through the machined support structure also caused the frequency difference between the two modes to become larger. Figure 9 also implies that the longitudinal and bending modes of the traveling wave vibrator were successfully excited.

**Figure 10:**
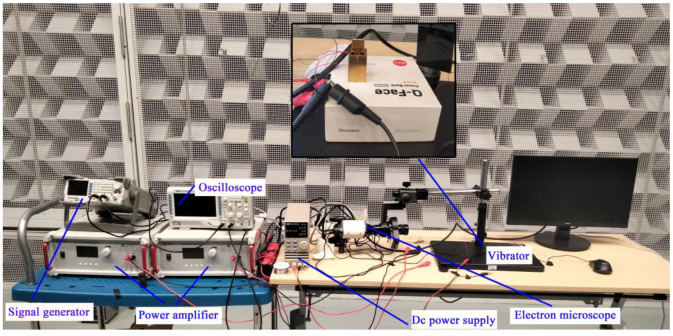
Experimental setup for testing the vibration responses of dust deposited on the surface of the vibrators.

Figure 10 shows the experimental setup used to drive the designed vibrators. During the experiments, the dust removal performance of the two types of vibrators was separately tested. Two-phase AC voltages were produced by a RIGOL DG1202 two-channel wave generator and amplified by two Aitek ATA-214 high-voltage power amplifiers. The frequency and phase difference of the excitation voltages could be adjusted by the signal generator, whereas the amplitude of the input voltages was mainly determined by the magnification of the amplifiers. The manufactured ultrasonic vibrators were fixed by the specially designed supporter. A RIGOL 1202 oscilloscope was used to observe the amplitude and phase difference of the two excitation voltage signals. The motion of dust was recorded using an AO-HD228SD electron microscope. This electron microscope can magnify dust particles up to 200 times.

**Figure 11:**
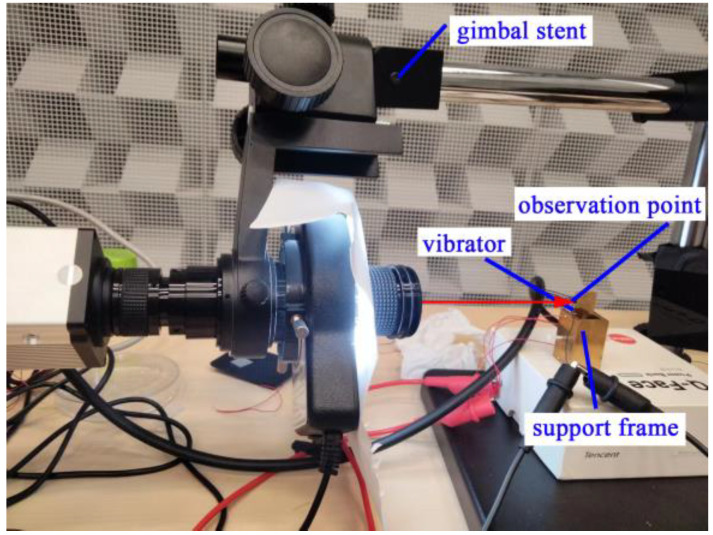
A partially enlarged view of the test component.

A gimbal stent, as shown in Figure 11, was added to detect the vibration of dust along the vertical direction. Vibrators were flexibly clamped using multiple Teflon screws of 2 mm diameter. Therefore, when we were observing the vibration of adhered dust particles, the ultrasonic resonators were macroscopically stable. The horizontal arrow in Figure 11 signifies the illumination direction of the electron microscope. It also indicates the inspection surface of the tested vibrator which was further enlarged and displayed on a viewing screen. Consequently, dust within the observation range of the vibrators could be clearly seen. Its micro-vibration properties could also be viewed. A dot was painted on the left side of the vibrator as a marker of the observation location. An HD228S camera was used to capture the motion of dust at 60 FPS/s with a 1920 × 1080 resolution. When the gimbal stent was removed, the beam of the electron microscope was oriented vertically. Then, the vibration of dust attached to the upper surface of the ultrasonic resonators could be observed.

**Figure 12:**
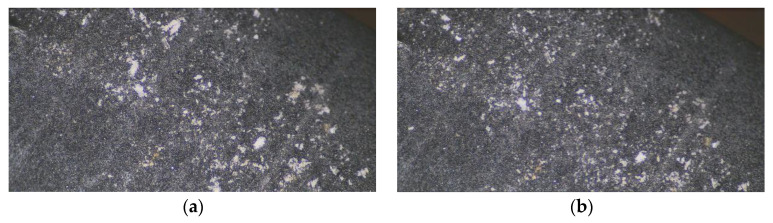
Comparison of dust particles on the vibrator surface: (**a**) Before vibration; (**b**) After vibration.

The voltage amplitude was set to 100 V, which was the same as that applied in the simulation analysis. It was assumed that the added support structure did not affect the resonant frequency of the working modes or the vibration of the ultrasonic vibrators. If the exciting frequency of applied voltages coincided with the resonance frequency of the longitudinal or bending mode, the standing wave vibrator started to oscillate according to the shape curve of the corresponding mode. Dust deposited on the surface of the vibrator moved along with it. In order to observe the vibration of dust on the whole surface of the oscillators, other surfaces of the oscillators without PZT plates were chosen as the observation area. Cigarette ash was selected as the observation object due to its easier obtainment and smaller dimensions. In addition, before observation, we used sandpaper to further rub the collected cigarette ash in order to get the smallest size of dust particles possible. Because of the relatively large observation area and the serious reflections from the brass, we pasted black tinfoil on the observation surface to reduce the reflections and increase imaging clarity. Figure 12a shows the initial state in which cigarette ash was deposited on the observing plane of the vibrators. The distribution of the cigarette ash on the vibrator surface when its vibration was stable is shown in Figure 12b. Whether the longitudinal or bending mode of the standing wave vibrator was excited by the actuation voltages, only some larger cigarette ash particles vibrated so violently that they were detached from the vibrator surface area. The experimental results also show that the dust removal efficiency of the standing wave oscillator under longitudinal or bending mode was approximately the same. The dust removal performance of the traveling wave oscillator was also measured by applying the same voltage amplitude. The frequency of the excitation signal was 32.28 kHz. However, the dust removal behavior of the traveling wave vibrator was not significantly improved. There were still many cigarette ash particles that could not be cleared. This is mainly because the bonded black tinfoil was not flat enough and particle deposition was not uniform. In Figure 12, it can be seen that some very small cigarette ash particles had fallen into tiny grooves on the surface of the black tinfoil and could not be further removed by ultrasonic vibration. Besides this, horizontally placed oscillators are not conducive to dust removal.

**Figure 13:**
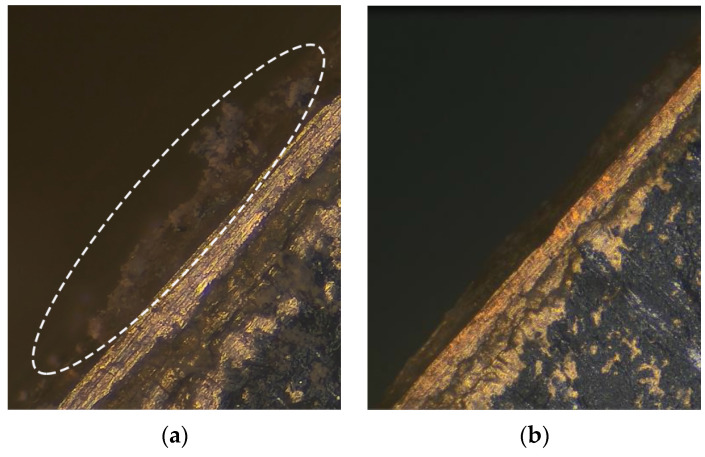
Contrasting distribution of dust particles on the left side of the vibrator: (**a**) Before vibration; (**b**) After vibration.

To facilitate the movement of dust, vibrators were placed vertically, as shown in Figure 11. In this case, the observation area was so small that it was not easy to paste black tinfoil. Therefore, we simply reduced the intensity of the illumination light. Cigarette ash particles were randomly sprinkled on the left side of the vibrators, as indicated by the blue dashed lines in Figure 13a. The voltage and working frequency were respectively set to 100 V and 32.28 kHz. Under the excitation frequency, the longitudinal and bending modes were excited simultaneously in the traveling wave vibrator. After a few seconds, the cigarette ash particulates on the observed traveling wave vibrator fell off completely with its vibration, as shown in Figure 13b. These results indicate that the prototype traveling wave vibrator can remove dust from its surface and prevent dust accumulation. However, the dust removal property of the standing wave oscillator was not obviously improved under this observation condition. Consequently, the dust removal nature of the standing wave oscillator is poorer than that of the traveling wave oscillator. The elliptical motion trajectory of the vibrator driving points of the traveling wave vibrator in the contact area is the key step in generating the driving force.

We also adopted 6 μm alumina powder to measure the dust removal characteristics of the devised ultrasonic vibrators. The vibration of some powder was observed clearly under the microscope. However, because alumina powder is smooth and hygroscopic, it easily adhered to the observation surface of the vibrators and gathered in bulk. Therefore, the effects of the ultrasonic vibrators on dust removal for alumina powder were not as good as those for cigarette ash.

## 5. Conclusions

In this paper, two kinds of ultrasonic vibrators were proposed to comparatively research their dust removal effects. These vibrators were constructed by bonding four PZT plates onto the upper surface of a metallic substrate with epoxy resin glue. The PZT plates vibrate within the XY plane with the d_31_ piezoelectric mode. The rectangular plate standing wave vibrator operates in longitudinal or bending mode, whereas the longitudinal and bending modes of the traveling wave vibrator are simultaneously excited. A traveling wave can be generated in the vibrator by superimposing two standing waves. This means that for the traveling wave vibrator, the resonance frequencies of the two operating modes should be kept close by setting its dimensions appropriately. The optimal structural parameters of the vibrators were obtained by modal analysis. The simulation results showed a discrepancy of 80 Hz between the two resonance frequencies of the traveling wave vibrator. The two modes form orthogonal vibration displacements on the vibrator driving points. The superposed elliptical motion of the vibrator particles produces friction force which drives deposited dust particles to move.

In order to evaluate the performance of the presented ultrasonic vibrators for the removal of microscopic dust particles, prototype vibrators were manufactured and assembled. PZT pates were connected to Aitek ATA-214 high-voltage power amplifiers which, in turn, were connected to a RIGOL DG1202 function generator. The vibration behaviors of cigarette ash placed on the surface of the prototype vibrators under their working modes were experimentally assessed. The experimental results suggest that, compared with the standing wave vibrator, the traveling wave vibrator has better dust removal characteristics. The developed traveling wave vibrator can effectively clear away fine particles of dust adhering to its surface. In addition, these vibrators do not require high-cost consumables, such as highly pure gases. Their simple structure, small size, low cost, and easy miniaturization ensure potential application prospects in dust removal from engines, camera lenses, car windows, and navigation systems. Since this dust removal method is realized by using the mode resonance of ultrasonic vibrators, the influence of target parts on the vibration of ultrasonic vibrators should be minimized when they are combined.

## Figures and Tables

**Table 1:** Material parameters of the PZT plates.

Piezoelectric Matrix (C/m^2^)	Dielectric Matrix (10^−9^ F/m)	Elastic Matrix (GPa)
$\begin{array}{cccccc} 0 & 0 & 0 & 0 & 14.3 & 0\\ 0 & 0 & 0 & 14.3 & 0 & 0\\ -4.9 & -4.9 & 18.5 & 0 & 0 & 0 \end{array}$	$\begin{array}{ccc} 8.496 & 0 & 0\\ 0 & 8.496 & 0\\ 0 & 0 & 7.680 \end{array}$	$\begin{array}{cccccc} 156 & 89 & 88 & 0 & 0 & 0\\ 89 & 156 & 88 & 0 & 0 & 0\\ 88 & 88 & 132 & 0 & 0 & 0\\ 0 & 0 & 0 & 34 & 0 & 0\\ 0 & 0 & 0 & 0 & 31 & 0\\ 0 & 0 & 0 & 0 & 0 & 31 \end{array}$

**Table 2:** Dimensions of the ultrasonic vibrators.

	Metal Base of the Standing Wave Vibrator			Metal Base of the Traveling Wave Vibrator				PZT Plates		
Parameter	L	W	T	L	W	T	D	L	W	T
Value (mm)	35	15	3	50	15	3	4	10	4	0.5

**Table 3:** Resonance frequencies of longitudinal and bending modes.

	Standing Wave Vibrator		Traveling Wave Vibrator	
Mode	Longitudinal	Bending	Longitudinal	Bending
Frequency (kHz)	51.15	57.42	31.95	32.03

**Table 4:** Comparison of the resonant frequencies of the two modes obtained by simulation and experiment.

	Standing Wave Vibrator		Traveling Wave Vibrator	
Mode	Longitudinal	Bending	Longitudinal	Bending
Frequency (kHz)	51.5	58.2	32.07	32.48
Deviation (%)	6.8	13	3.7	13.8
